# Lipid MRI in plant science: principles and potential areas of application

**DOI:** 10.1093/jxb/eraf479

**Published:** 2025-10-28

**Authors:** Ljudmilla Borisjuk, Thomas Neuberger, Hardy Rolletschek

**Affiliations:** RG Assimilate Allocation and NMR (AAN), Leibniz-Institute of Plant Genetics and Crop Plant Research (IPK), Seeland-Gatersleben 06466, Germany; Huck Institutes of the Life Sciences, Pennsylvania State University, University Park, PA 16802, USA; Department of Biomedical Engineering, The Pennsylvania State University, University Park, PA 16802, USA; RG Assimilate Allocation and NMR (AAN), Leibniz-Institute of Plant Genetics and Crop Plant Research (IPK), Seeland-Gatersleben 06466, Germany; Donald Danforth Plant Science Center, USA

**Keywords:** Artificial intelligence, lipid imaging, magnetic resonance imaging, NMR spectroscopy, oilseed phenotyping, post-transcriptional regulation, spatial heterogeneity

## Abstract

Magnetic resonance imaging (MRI), long established in medical diagnostics, offers powerful, non-invasive capabilities for visualizing physiological processes in intact plants. This review focuses on the principles, recent advances, and future prospects of MRI-based lipid analysis in plant science with a particular focus on seeds. Cutting-edge, spatially resolved MRI has uncovered a remarkable compartmentation of lipid metabolism and storage. Lipid distribution patterns reflect the tissue- and cell-specific functional roles of lipids and are shaped by local metabolite gradients and other regulatory factors, including biomechanical and environmental stimuli. Recent innovations in MRI methodology now allow comprehensive, non-invasive monitoring of lipid storage and degradation dynamics *in vivo*. Looking ahead, the integration of MRI with deep learning and multimodal approaches heralds a transformative era for seed biology, oilseed phenotyping, and breeding.

## Teaser

This article outlines the key strategies of MRI-based lipid imaging, the latest methodological advances, and applications in plant science with a focus on seeds.

## Introduction

‘The science of today will be the technology of tomorrow.’

Edward Teller, theoretical physicist (1975)

Lipids fulfil a wide range of vital roles in plant life. They constitute the architectural framework of cellular membranes, contribute to the formation of protective barriers and serve as an energy store. They are also involved in signalling and adaptation to a changing environment. As attention grows on the intricate ways in which lipids influence plant physiology (Suh *et al*., 2022), the localization and dynamic analysis of lipids within plant tissues has emerged as an increasingly significant field. Insights into the biological functions of specific lipid classes in different cell types and tissues could be provided, shedding light on their roles in development and stress responses. This knowledge could significantly advance agricultural practices and industrial applications, leading to higher-yielding and more resilient crops.

Which tools are required for a comprehensive understanding of lipid function in plant tissues? The ideal approach would be to use high-resolution, *in vivo* imaging to enable the quantitative, label-free monitoring of lipids in living plants. It would also be highly desirable to co-visualize lipids alongside metabolites, proteins, or RNAs, preferably through the use of one versatile imaging platform. While the field of scientific imaging is undergoing rapid transformation and making great strides towards achieving this goal, there is still some way to go.

Conventional light microscopy offers a variety of methods, including the use of various lipophilic dyes (like Red O and Sudan Black) and fluorescent dyes (like Nile Red and Boron-dipyrromethene). These methods are usually semi-quantitative and efficient when applied to tissue sections and/or fixed tissues. Infrared (IR)-based microscopy, such as vibrational spectroscopy, detects lipid-specific absorption bands, enabling the rapid visualization of lipids in tissue sections without the need for labels (Borisjuk *et al*., 2023). Modern label-free methods rely on specifically stimulated Raman scattering (SRS) and third-harmonic generation (THG) microscopy to determine the molecular identity and physical structure of lipid assemblies within cells (Débarre *et al*., 2006; Kim *et al*., 2024). However, overlapping spectral features can limit chemical specificity, particularly when studying bulk tissues, thereby limiting their applicability to living plants. Alternatively, genetically encoded biosensors can be considered for use in studying lipid dynamics *in vivo* for species with genetic transformation systems (Li *et al*., 2025). Mass spectrometry imaging is the most informative yet invasive method as it enables the chemical resolution and mapping of hundreds of lipid species in tissue sections (Sturtevant *et al*., 2021).

A range of X-ray technologies have been utilized in the field of seed research and can be adapted for the investigation of lipids; however, chemical resolution has remained constrained (Ashe *et al*., 2025). The utilization of contrast agents, accompanied by their purported toxicity, has the potential to supersede the benefits associated with non-invasiveness. Positron emission tomography (PET) and autoradiography are analytical techniques capable of measuring the three-dimensional (3D) spatial distribution and kinetics of radio-tagged biomolecules in a living organism (Komarov and Tai, 2022). Therefore, the utilization of radiolabelled tracers and lipid precursors can be employed to trace lipid biosynthesis, allocation, and turnover (Lundström *et al*., 2021).

The magnetic resonance imaging (MRI) approach is distinct from alternative methods in that it utilizes nuclear magnetic resonance (NMR) principles, thereby facilitating deep tissue penetration without the necessity for staining or destructive sampling. It is generally non-invasive, label-free, and capable of providing 3D spatial resolution (Borisjuk *et al*., 2023). In other words, it enables researchers to visualize structures, to image lipid distribution and dynamics, and to quantitatively monitor oil accumulation and degradation processes. The present review builds on a series of seminal works that established the foundations of MRI-based lipid imaging in plants (Neuberger *et al*., 2008, 2009; Borisjuk *et al*., 2013a; Munz *et al*., 2016). We provide an overview of available MRI methods for lipid analysis, highlight cutting-edge applications in oilseed biology, and discuss the biological factors underlying lipid heterogeneity within seeds as revealed by MRI and complementary approaches. Finally, we share our perspective on recent advances in lipid imaging and outline promising directions for future applications.

## The physics behind NMR imaging

### From ^1^H spin to lipid signal

**Fig. 1.**
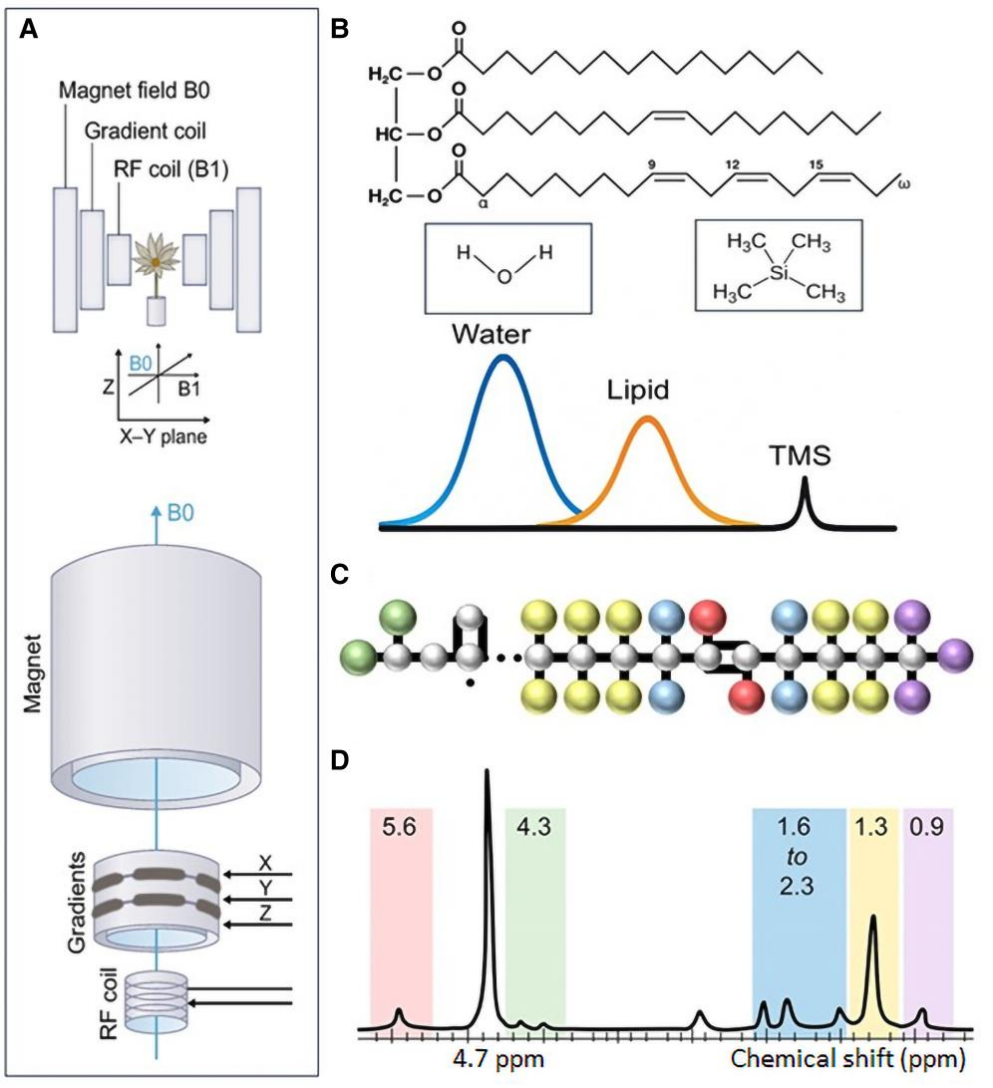
Experimental set-up and interpretation of the ^1^H NMR spectrum. (A) Schematic of the MRI scanner and position of the specimen within the bore; the upper section shows the orientation of the plant specimen within the strong static magnet field B0 and the radio frequency (RF) field B1 (mid-section); the lower section illustrates the compounds of the main compounds of the vertical bore MRI microscope. (B) Top panel shows water, lipid (TAG) and tetramethylsilane (TMS). Low panel schematically shows a 1D proton spectra with the relative positions of TMS as the reference at 0 ppm, a lipid peak, and water at 4.7 ppm. (C) Scheme shows a fragment of a lipid molecule made up from carbon (white) and hydrogen (variegated). (D) ^1^H NMR spectrum (simplified) displays the signals acquired from protons of the displayed lipid molecule. The NMR frequency of the signal of protons varied corresponding to their molecular environment, as shown by the same colour code. Red: single bondHCdouble bondCHsingle bond; green: glycerol backbone; blue: (CH2)n–CH2–HCdouble bondCH, CH2single bondCOOH, CH2single bondCH2COOH; yellow: (CH2)n; purple: (CH2)n-CH3. The water peak appears at 4.7 ppm. The intensity of the individual peaks is informative for quantitative interpretation of the spectrum.

To create an MRI image, the sample is placed in a homogeneous magnetic field (B0), measured in tesla (T), and generated by the main magnet of the system (Fig. 1A). The experimental design for plants is not constrained to a certain field strength and can leverage a wide range of systems operating from 50 μT to 23.5 T (Borisjuk and Neuberger, 2025). Once placed in the magnetic field, the spin of the NMR-active nuclei within the specimen start to precess at a field-dependent frequency and start to align slightly with the main magnetic field B0 which coincides with the z direction of a coordinate system. Over time (characterized by the spin lattice relaxation time T1) a magnetization is building up exponentially until a net magnetization (M₀) within the specimen is reached. This maximum magnetization can now be tilted towards the xy-plane using a linear or circular polarized magnetic field (B1-field) created by a radio frequency (RF) coil (Gruber *et al*., 2018). The in the xy-plane precessing magnetization (Mxy) is losing its amplitude over time; it is said that the magnetization is slowly decaying exponentially. The decay is characterized by the spin-spin or T2 relaxation time. Before this magnetization is perishing, it will be recorded with the same or an additional RF coil comprising our MR signal. As different tissues possess different T1 and T2 times, varying the time between the RF pulses (repetition time, TR) and changing the time from excitation to acquisition (echo time, TE) allows to manipulate the contrast in the MR image. Besides these two approaches, the spin density plays a major role in image contrast. To achieve spatial localization of the signal in two or three dimensions three orthogonal linear changing magnetic field gradients are applied in certain patterns resulting in different frequencies of the Mxy at each location. This procedure is the main difference to most other imaging techniques. The image is not acquired in an actual image space but in the so-called Fourier space. After applying a 2D or 3D inverse fast Fourier transformation, the image is finally revealed.

The resonance frequency *ω*_0_ of a proton’s spin in a magnetic field is directly proportional to the product of the nuclear gyromagnetic ratio *γ* (an atom specific constant) and the external field strength *B*0 and is called the Larmor frequency:


(1) $$\omega_{0}=\gamma \cdot B0$$

As atoms experience different chemical environments depending on their position within a molecule, their resonance frequency can slightly be altered or shifted. This so-called chemical shift (*σ*) is mainly produced by electron shielding of the main magnetic field. The shift can be a few parts per million (ppm, 1 ppm @ 7T is equal to 300 Hz) in hydrogen (^1^H) MRI or more than 100 ppm in ^13^C MRI. The Larmor equation (equation 1) with the chemical shift considered reads:


(2) $$\omega_{0}=\gamma \cdot B_{o}(1-\sigma ).$$

In ^1^H MRI, the resonance frequency of tetramethylsilane (TMS) is used as a reference frequency and set to zero (Fig. 1B). Protons in water show a higher shift (∼4.7 ppm) than those in lipids [∼1.2 ppm for (CH₂)ₙ]. While the use of ppms allows identifying the specific protons independently of *B*₀, the move to higher *B*₀ will increase the absolute difference between e.g. water and lipids in Hz. This increased spectral resolution enables a better distinction of specific protons within a molecule at higher fields. Therefore, compounds with spin-active nuclei can be identified based on their characteristic *σ* values or resonance frequency (Fig. 1B–D). Lipid molecules are composed of a significant proportion of reduced carbon atoms, which bind with a substantial number of hydrogen atoms. These can be readily detected through the utilization of proton (^1^H) and carbon (^13^C) nuclear magnetic resonance (^1^H NMR and ^13^C NMR). However, ^13^C NMR is approximately 100–150 times less sensitive than ^1^H NMR, and the natural abundance of ^13^C is only ∼1.1% of all carbon atoms. Consequently, although lipids can be detected by ^13^C NMR, this generally requires concentrated or extracted samples, long acquisition times, or isotopic enrichment. These limitations render the investigation of lipids by ^13^C MRI generally impractical.

### Magnetic resonance approaches for lipid detection in plants

Historically, the earliest NMR experiments were non-localized, capturing bulk signals from entire samples. This approach laid the conceptual foundation for modern magnetic resonance techniques, including MRI. Beginning with these origins underscores the transition from bulk to spatially resolved methods and provides a complete view of the methodological spectrum of magnetic resonance in plant science.

The simplest not localized procedure for obtaining a spectrum is referred to as the continuous-wave (CW) method. This approach applies a constant (continuous) RF field while sweeping the magnetic field or RF frequency, and measures absorption of RF energy in real-time as the resonance condition is met. Therefore, it yields an average signal from the whole sample and accordingly represents the global spectral profile of the sample. This method is very fast and needs only milliseconds, allowing detection of both water and lipids—or selective detection of one—depending on the excitation pulse parameters. These measurements do not necessarily require high magnetic fields or gradient fields. Global spectroscopy is well suited for rapid sample analysis, such as seed screening. Continuous-wave ^1^H NMR has been used to non-invasively quantify lipids in up to 24 000 seeds per hour, with measurement times of approximately 150 ms per seed (Colnago *et al*., 2007). Another not localized procedure is the application of a RF pulse and the following acquisition of the MR signal. Depending on the bandwidth and the frequency of the RF pulse, spectra from water only, lipid only, or the combination of both could be acquired. Global spectroscopy provides no localization information and is therefore not suitable for studies that require spatial resolution. However, the principle of non-localized bulk measurement lives on in pulsed global spectroscopy (single-pulse free induction decay acquisitions).

Pulsed field gradient (PFG) techniques are now the standard for MRI and have been used as an alternative to phase cycling for many years (Barker and Freeman, 1985). These techniques use the aforementioned three orthogonal linear magnetic field gradients to produce different local frequencies within the desired imaging volume. When combined with a fast signal acquisition system, this approach generates spatially resolved images; images of lipids or water only can be produced in ^1^H imaging. Various applications including the analyses of storage lipid accumulation *in planta* during development and the estimation of oil bodies sizes have been successfully demonstrated (Annarao *et al*., 2008; Gromova *et al*., 2015).

Localized spectroscopy is another approach to detect lipids in plants. With this technique information on lipid content of a selected voxel (short for volume element) anywhere within the plant organ or general region of interest can be obtained. To achieve localization, this method uses frequency selective RF pulses in combination with the magnetic field gradients. The acquired spectrum from the selected region displays the chemical composition present in the selected voxel. Point-resolved spectroscopy (PRESS) and stimulated echo acquisition mode (STEAM) are the two main localized spectroscopic methods. They both utilize three RF pulses in combination with specific gradients. The first RF pulse in combination with the gradient excites a distinct plane. While the second RF pulse and orthogonal gradient excites an endless ‘pencil shape’ within this plane, the third RF pulse and the third orthogonal gradient finally only excite a voxel. While STEAM has certain advantages the PRESS sequence produces two times more signal-to-noise ratio and is therefore preferred when low lipid (metabolite) concentrations are present. These sequences are often combined with a water suppression module [CHEmical Shift Selective saturation (CHESS) or VAriable Power radiofrequency pulses with Optimized Relaxation delays (VAPOR)] as, especially in younger or low lipid containing plants, water has a much higher concentration, and the lipid signal would not be seen. PRESS has been used to monitor lipid accumulation in *Areca catechu* embryos (simultaneously measuring water, sugar, and alkaloid content; Srimany *et al*., 2016). For other metabolites with fast T₂ decay (like sugars or amino acids) STEAM might be more suitable.

### The main strategies for high resolution lipid imaging

**Fig. 2.**
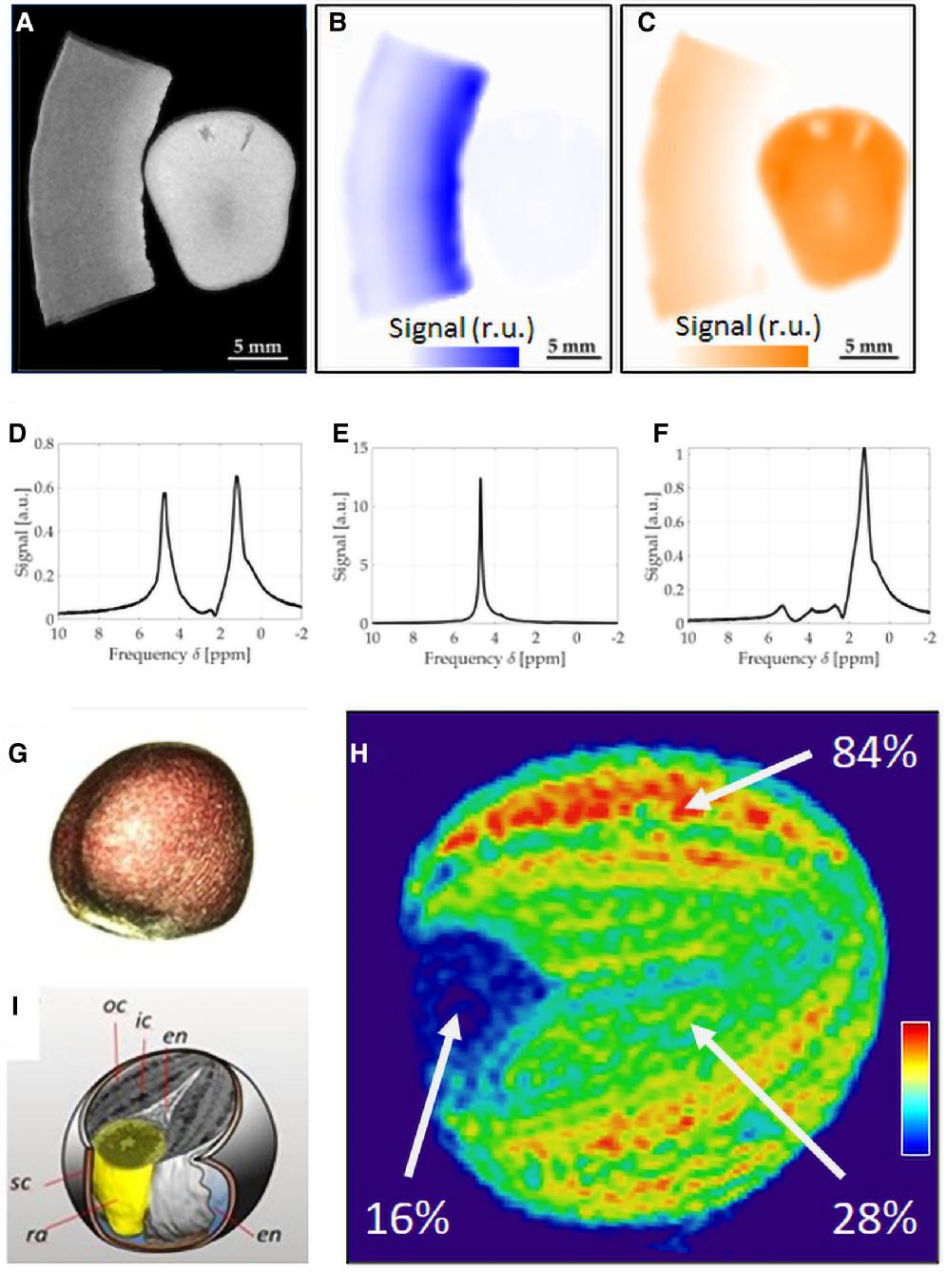
MRI analysis of lipid distribution in mixed and complex samples. (A–F) MRI of the mixed sample consisting of the freshly hand-cut coconut endosperm (left) and dried Brazil nut kernel (right) at 11.75 T. (A) Structural MR image. (B) CSI method shows the water distribution in fresh coconut endosperm, with a clear gradient from the inner to the outer region, whereas the dried Brazil nut kernel remains invisible. (C) CSI with water suppression shows the lipid distribution in both samples, revealing a pronounced gradient in the coconut endosperm and a weaker one in the Brazil nut kernel. (D) Localized CSI spectrum (measured without water suppression) of coconut endosperm showing signals of water (4.7 ppm) and lipids (1.2 ppm). (E) Spectrum from inner region of coconut endosperm showing only water. (F) CSI spectrum of the Brazil nut kernel dominated by lipid signals, including resonances of saturated (1.2 ppm) and unsaturated (5.2 ppm) fatty acids (courtesy of S. Mayer, IPK/Germany, see Supplementary Protocol S1). (G–I) Lipid gradients in mature seeds measured using CSSI at 17.5 T. (G, I) A photographic image of the oilseed rapeseed (*Brassica napus*) and a schematic drawing showing the arrangement of seed tissues. The image in (H) shows a colour-coded lipid map, which is a virtual cross-section of the seed. The arrows indicate regions with clearly different storage oil levels, ranging from 16% in the radicle to 84% in the outer cotyledon. Reproduced with permission from Borisjuk *et al*. (2013a). Abbreviations: en, endosperm, ic, inner cotyledon, oc, outer cotyledon, ra, radicle, sc, seed coat.

There are two main approaches to image lipids with a high spatial resolution (Fig. 2). The first one is chemical shift imaging (CSI) and the second one is chemical shift selective imaging (CSSI).

CSI acquires spectra from many voxels simultaneously and uses the three orthogonal magnetic field gradients to spatially resolve these voxels. As every single voxel in this technique contains a full spectrum, certain frequency ranges with signals from metabolites of interest can be selected and metabolite maps can be reconstructed (Fig. 2A–F, see also Supplementary Protocol S1). CSI has been used together with other MRI protocols to explore the types of information retrievable from specimens which are difﬁcult to investigate by conventional methods (Glidewell *et al*., 2002). Compared to regular imaging sequences which acquire a whole line in Fourier space per excitation pulse, no gradient is present during data acquisition of the CSI sequence, resulting in the acquisition of only one point in Fourier space for each excitation. This limitation results in relative long acquisition times, especially when data is acquired in 3D. If averaging to achieve a higher signal-to-noise ratio is necessary certain techniques like accumulation weighted CSI (Pohmann *et al*., 1999) can be applied to reduce the scan time.

**Fig. 3.**
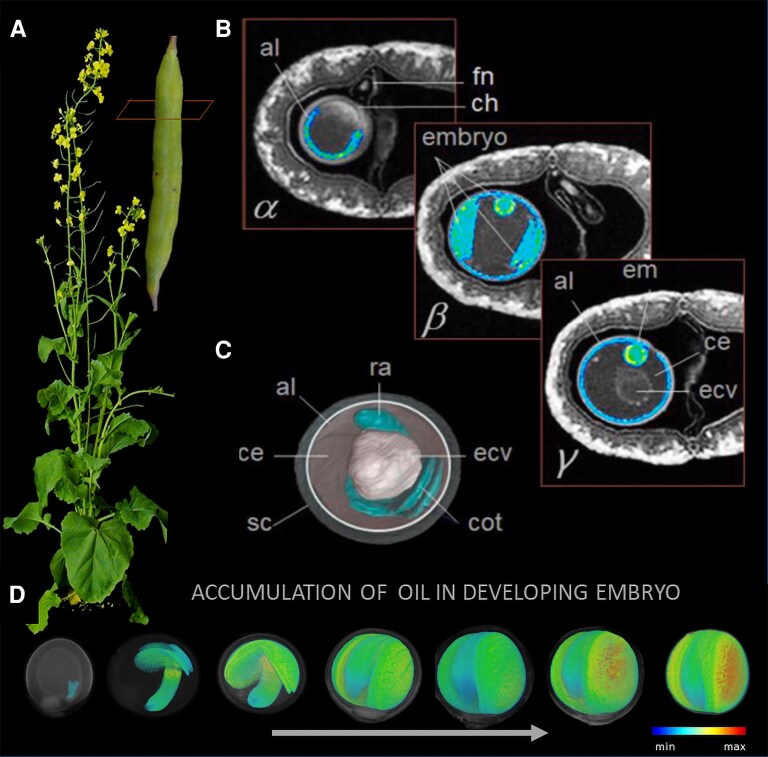
MRI-based visualization of tissue-specific lipid storage in developing embryo of oilseed rapeseed (*Brassica napus*). (A) Optical image of rapeseed plants at flowering and seed set; also shown is a typical silique, which contains seeds. (B) Virtual cross-sections through the silique displaying spatial arrangement of seed components at the mid-cotyledon stage of development; MR-images indicate lipid accumulation (colour-coded) in the aleurone layer of endosperm and in the embryo proper. Alpha, beta, and gamma show successive sections through a single seed. (C) Three-dimensional seed model showing all individual seed components. (D) Three-dimensional MRI showing the developmental sequence of oil deposition, starting from the early cotyledon stage of embryogenesis; lipid levels are colour-coded with min (max) lipid signal intensity given in blue (red). Reproduced from Rolletschek *et al*. (2021) under a Creative Commons CC-BY license. Abbreviations: al, aleurone; ce, cellularized endosperm; ch, chalazal region; cot, cotyledon; ecv, endosperm central vacuole; em, embryo; fn, funiculus; ra, radicle; sc, seed coat.

**Fig. 4.**
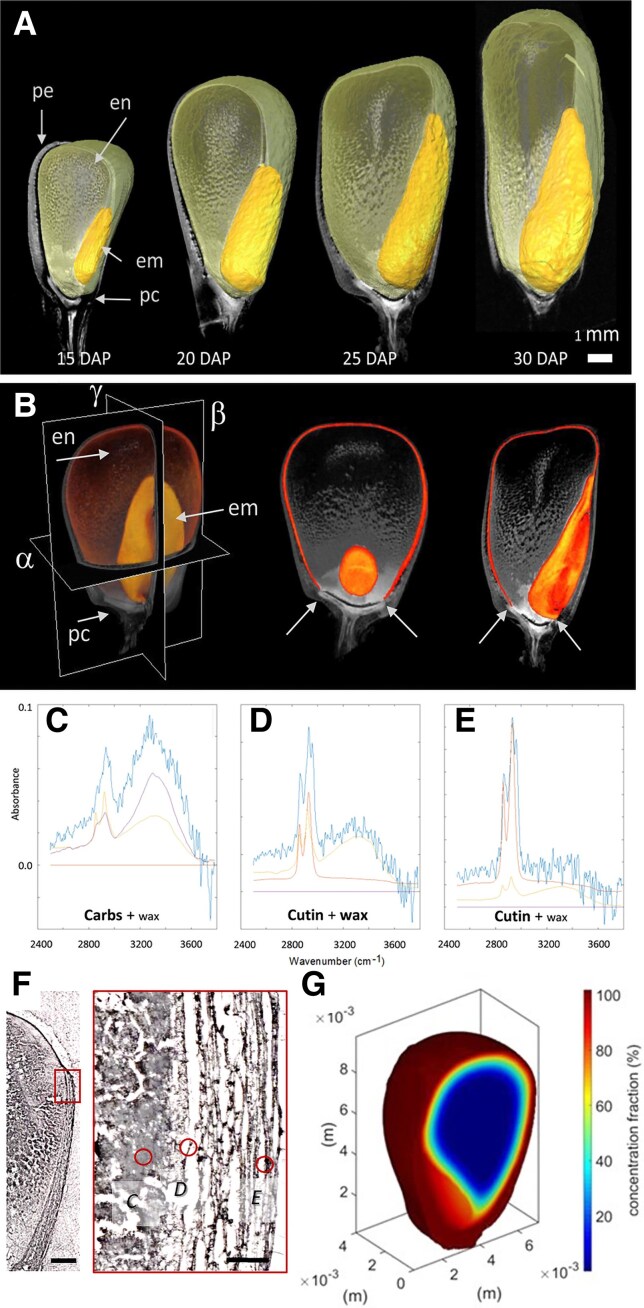
Localization and chemical features of lipid layers covering the endosperm of developing maize (*Zea mays*) kernels. (A) MRI-based structural modelling of developing maize kernels at 15, 20, 25, and 30 days after pollination (DAP); the three-dimensional models reveal the location and expansion of the embryo and the endosperm during development. (B) MRI-based lipid mapping indicating the presence of lipid at the endosperm surface and in the embryo; 3D scheme indicates the virtual sections along the β plane (middle panel) and γ plane (right panel); arrows indicate the lack of lipidous layers at the basal endosperm region; all images were digitally extracted for comparison, and use the same scale bar. Lipid signal is given in orange. (C–F) Chemometric analysis of micro-ATR image spectra of maize kernels taken in the region of (C) starchy endosperm, (D) endosperm surface layer, and (E) outer epidermis of pericarp. Spectral panels in (C–E) present the measured spectrum at the given location in blue, and superimposed contributions of cutin (red), wax (yellow), and carbohydrate (purple, carbohydrate) library spectra. The dominant spectral feature is highlighted in bold letters. Left panel of (F) gives a partial view image of a cryo-sectioned maize kernel; a red box locates the analysed region for ATR imaging; the magnified image in the right panel depicts the location of the analysed spectra shown in (C–E). (G) Model of oxygen concentration in a longitudinal plane, indicating most severe oxygen deficiency (hypoxia) in the major part of the starchy endosperm. Model was generated based on X-ray CT. All images reproduced with permission from Langer *et al*. (2023). Abbreviations: en, endosperm; em, embryo; pc, pedicel; pe, pericarp.

CSSI is the method of choice when an experiment targets only one or a few specific compounds and requires high-resolution images of those metabolites within a moderate measurement time (Neuberger *et al*., 2009) (Fig. 2G–I). CSSI is very similar to standard imaging but selectively excites only the resonance frequencies of interest, allowing for direct imaging of specific metabolites. Unlike CSI, this approach acquires a whole line in Fourier space per excitation and is therefore significantly faster. It produces metabolite-specific images without the need for spectral decomposition, offering better signal-to-noise ratio per image and resulting in smaller, more manageable datasets (Figs 2H, 3B, D, 4B). There are three main strategies for applying CSSI to visualize lipids in plants:

- Regular spin-echo imaging without selection or suppression: this approach uses a basic spin-echo sequence without the need for frequency selection or water suppression and is applicable when lipids are the only detectable NMR-active component, such as in dry mature seeds or other dehydrated tissues. In these cases, the signal from bound water decays too quickly (short T_2_) to contribute meaningfully to the signal, allowing imaging to proceed with minimal preparation. This method has achieved the highest in-plane resolution reported for plant lipid imaging and has been successfully applied to seed screening, comparative analysis of freeze-dried materials, and other purposes (Terskikh *et al*., 2008; Fuchs *et al*., 2013)
- Selective suppression of dominant signals: this approach is used when the lipid signal is masked by a dominant NMR signal from another component, typically water. In this approach, the dominant signal is selectively suppressed using one or more frequency-selective RF pulses in combination with pulsed field gradients, applied just before the actual imaging sequence. The RF pulses flip the longitudinal magnetization (Mz) into the xy-plane and the applied gradient pulses destroy the coherence of the transverse magnetization of the dominant signal. As only a short delay separates the suppression module from the imaging sequence, recovery of magnetization along the z-axis (governed by the T1 relaxation time of the dominant signal) is negligible. Commonly used suppression modules include CHESS (Haase *et al*., 1985) and VAPOR (Tkáč *et al*., 2021). By applying such selective suppression, interfering signals can be reduced or eliminated, thereby allowing clearer detection and analysis of the compound of interest. This technique enables selective imaging of lipids by suppressing water, or vice versa (Hong *et al*., 2009; Borisjuk *et al*., 2013b).
- Frequency-selective excitation at ultra-high magnetic fields: due to the large spectral resolution at ultra-high magnetic fields, CSSI can incorporate frequency-selective excitation and refocusing pulses that excite only single metabolites. As mentioned before, in ^1^H MRI, different chemical groups resonate at distinct frequencies (chemical shifts), enabling a selective excitation of lipid protons such as –CH₂– groups around 1.2 ppm while water signals near 4.7 ppm are not touched at all. This allows for direct imaging of lipids with minimal interference from water or other tissue components (Neuberger *et al*., 2008; Borisjuk *et al*., 2013a; Munz *et al*., 2016).

CSSI has been applied in various studies on fruit ripening (olive: Brescia *et al*., 2007) and seed development across a wide range of plant species. It has been successfully used on grains with low oil content—such as barley, wheat, and maize (Fig. 4B;  Kovalchuk *et al*., 2016; Langer *et al*., 2023)—as well as on seeds with high oil content, including soybean (Borisjuk *et al*., 2005), oat (Hayden *et al*., 2011; Rolletschek *et al*., 2015), oilseed rape (Fig. 2H, 3B, D;  Rolletschek *et al*., 2020, 2021, 2024), cotton (Horn *et al*., 2012), and *Camelina sativa* (Horn *et al*., 2013). The technique is capable of producing quantitative lipid maps with near-cellular resolution (tens of microns). Its reliability has been validated by comparison with conventional analytical methods, including gas chromatography and mass spectrometry imaging, making it a valuable tool in experimental botany and breeding research.

### Lipid analysis using low-field NMR is constrained by certain factors

Imaging of lipid distribution at low magnetic field strengths using bench-top systems has remained very challenging to date. However, there is a growing interest in portable and low-cost devices for outdoor experiments. Low-field MRI has certain advantages, such as different relaxation times and reduced susceptibility artefacts, compared to high (>3 T) or ultra-high (>9.4 T) field strengths. In clinical imaging, low-field MRI has recently received considerable attention (Marques *et al*., 2019; Liu *et al*., 2021) and is sometimes the preferred method. For instance, imaging the lungs using high-field MRI is difficult due to the numerous air-tissue interfaces. These cause strong magnetic susceptibility changes, field inhomogeneities, signal loss and, eventually, image distortion. Therefore, 0.5 T systems might be more efficient for lung MRI, whereas higher fields are preferred for organs such as the brain. In plant research, low-field time-domain nuclear magnetic resonance (TD-NMR) systems use relaxation parameters (T₁ and T₂) to estimate chemical content (Colnago *et al*., 2021). These systems offer simple sample preparation, fast assays, and high-throughput analysis. However, their low signal-to-noise ratio (i.e. low sensitivity) and reduced frequency separation between water and lipids render them unsuitable for precise lipid imaging in water-rich tissues. Other sample properties, such as matrix composition and the state of the lipids (liquid versus solid), may also influence the NMR signal. The trade-off between cost efficiency, simple sample preparation, and high throughput versus limitations in sensitivity and resolution should be carefully evaluated in each case. Low-field MRI has been used in various lipid screenings [e.g. maize kernels: Kotyk *et al*. (2005); oil palm fruits: Shaarani *et al*. (2010); cassava seeds: Iulianelli and Tavares (2016); oats: Rolletschek *et al*. (2015)]. When properly calibrated and optimized, low-field MRI is a powerful tool for non-destructive, quantitative lipid screenings, particularly in plant breeding, seed quality control, and industrial oil processing. However, lipid imaging at low-field strength is still not widely available and requires further technological development.

## Potential areas of applications for lipid MRI

### Relevance of non-invasive approaches for study of ancient functions of lipid storage in cells

Lipids are essential for the structure and function of almost all living organisms, and they have conserved roles across eukaryotes. One of their key roles is to store energy, primarily in the form of neutral lipids such as triacylglycerols (TAGs) and sterol esters. These energy-rich molecules are stored in lipid droplets (LDs), which are specialized organelles surrounded by a phospholipid monolayer and associated proteins (Guzha *et al*., 2023). A serendipitous early observation of intracellular lipids using MRI came from imaging a *Xenopus laevis* ovum at 10×13×250 µm resolution with a 1.5 T clinical scanner, where lipid utilization was observed during the experiment (Aguayo *et al*., 1986).

LDs occur throughout the plant kingdom, from liverworts and mosses to higher plants, but their abundance and function vary across tissues. In vegetative tissues, for example, LDs are typically sparse. In photosynthetic tissues, their abundance can fluctuate diurnally (Gidda *et al*., 2016); in roots, they are associated with specialized processes such as tuber formation in *Cyperus esculentus* (Turesson *et al*., 2010). In the buds of perennial plants such as Norway spruce (Guzicka *et al*., 2018), aspen, and poplar (Veerabagu *et al*., 2021), LD levels increase during winter dormancy (Sutinen *et al*., 2012; Bouchnak *et al*., 2023). LDs are especially abundant in seeds, which are evolutionarily tasked with plant survival and dispersal (Shimada *et al*., 2018; Ischebeck *et al*., 2020).

LD and TAG accumulation also occur under abiotic stress, including heat, drought, and cold. In *Arabidopsis thaliana*, membrane-derived acyl chains are redirected into TAGs, likely serving as a transient metabolic sink (Mueller *et al*., 2015). Similar stress-induced LD accumulation has been seen in stress-exposed chlorophyte algae (Li-Beisson *et al*., 2019). Clearly, LDs are more than inert oil stores—they are multifunctional organelles involved in development, stress adaptation, and metabolism (Ischebeck *et al*., 2020; Lundquist *et al*., 2020). The formation of LDs under stress appears to be a conserved response across plant lineages, raising a compelling evolutionary question: is the accumulation of LDs and TAGs during seed development simply a specialized version of this ancient stress-response mechanism?

De Vries and Ischebeck (2020) argue that the relationship between stress and LD formation pre-dates the evolution of seeds. They propose an evolutionary scenario in which (i) LD formation originally evolved to enhance resilience against drought and desiccation, and (ii) the necessary protein framework was later co-opted by seed plants (spermatophytes) for specialized lipid storage during embryogenesis. In this view, the seed-specific lipid storage programme represents an adaptation of a more ancient, stress-related cellular strategy—one that is now traceable and quantifiable through *in vivo* imaging techniques such as MRI.

MRI enables the imaging of submillimetre-sized seeds, such as those of *Nicotiana tabacum* (Fuchs *et al*., 2013), and allows for non-invasive quantification (Neuberger *et al*., 2008, 2009) and monitoring of lipid dynamics during seed development and germination (Munz *et al*., 2017). Due to current limitations in magnetic field strength and scan time, the resolution of MRI is generally constrained to approximately 1 µm (Glover and Mansfield, 2002). Although MRI does not match the resolution capabilities of conventional light or electron microscopy, its strength lies in the non-destructive nature and capacity to visualize internal structures and dynamic physiological processes *in vivo*. These modalities offer complementary insights that traditional, invasive techniques cannot provide.

### An MRI perspective on the natural capacity of oil storage and its engineering in plant tissues

Seeds and fruits are the main sources of vegetable oils. TAG biosynthesis in seeds occurs in specialized storage tissues with critical developmental roles, making oil trait engineering challenging (Weselake *et al*., 2024). In rapeseed, lipid content varies markedly between tissues, with the embryo holding far more oil than the seed coat or endosperm, and over a two-fold difference between the radicle and cotyledons (Fig. 2G–I). Understanding spatial heterogeneity in oil accumulation is essential for identifying regulatory mechanisms and optimizing crop oil traits. MRI enables high-resolution, non-destructive, and quantitative mapping of lipid distribution, supporting precise phenotyping and rapid screening of germplasm, examples of which include rapeseed (Borisjuk *et al*., 2013b), tobacco (Fuchs *et al*., 2013), oat (Rolletschek *et al*., 2015), and barley/wheat (Plutenko *et al*., 2025).

A well-documented phenomenon in seed biology is the negative correlation between oil and protein content in seeds, suggesting a competitive relationship between carbon and nitrogen allocation. Although efforts have been made to shift metabolic fluxes from protein to lipid biosynthesis (or vice versa), practical outcomes have been limited. For instance, in transgenic *Brassica napus* with nearly 90% repression of the main storage proteins (napin and cruciferin), seed oil levels and phenotype remained nearly unchanged (Rolletschek *et al*., 2020). However, comprehensive multi-modal analyses uncovered a remarkable plasticity in embryonic cell structure and metabolism.

Oilseed engineering often modifies the qualitative composition of seed oil, such as increasing the proportion of unsaturated fatty acids or incorporating rare and exotic fatty acids. These modifications, however, can have unintended consequences on seed metabolism and development, which warrant careful investigation (Horn *et al*., 2013; Bashiri *et al*., 2024). For example, enhanced accumulation of wax esters has been associated with reduced germination (Zhu *et al*., 2016; Ivarson *et al*., 2017; Sturtevant *et al*., 2020). Currently, MRI does not resolve fatty acid composition, but can be applied in combination with magnetic resonance spectroscopy to be developed for such applications.

Efforts are made to engineer lipid accumulation in vegetative tissues for biofuel production. Tobacco plants engineered to accumulate TAGs in leaves exhibited major shifts in carbon partitioning, with downstream effects on biomass and plant development (Vanhercke *et al*., 2014; Mitchell *et al*., 2017). While enhancing lipid content holds great promise, it may entail trade-offs in growth and vigour—highlighting the need for comprehensive investigation of progeny. MRI offers unique advantages in this context, allowing the detection of oil accumulation in any plant organ and providing insights into associated structural and metabolic changes *in vivo* (Borisjuk and Neuberger, 2025). As such, it holds significant potential to support precision breeding and metabolic engineering aimed at improving oil yield and composition.

While conventional tools for seed screening focus on external seed parameters or 2D imaging, they fail to capture internal tissue architecture and composition without dissection (He *et al*., 2022; Jayakodi *et al*., 2023). Alternatively X-ray computed tomography is applied, but often fails to identify tissues with similar electron density (Le *et al*., 2019; Zhou *et al*., 2021). MRI offers unique advantages over X-ray, particularly for visualizing soft tissues and metabolites like lipids *in vivo*, without the need for ionizing radiation or contrast agents. This makes MRI especially suitable for seed screening, enabling structural and metabolic insights that are critical for modern breeding and biotechnology (Plutenko *et al*., 2025).

### MRI insights into the barrier role of lipids in seed compartmentation

While the outermost layers of the mature seed—such as the seed coat—form a sealed interface that protects the embryo from the external environment, structurally distinct lipid-containing internal barriers are also commonly found within the seed interior. These internal barriers are often semi-permeable, regulating the movement of water, gases, and nutrients. Their collective action is protective and critical in preventing damage and enabling seeds to survive under adverse environmental conditions in a metabolically inactive state (with water content below 5%) (Radchuk and Borisjuk, 2014; Rolletschek *et al*., 2025).

The structure and organization of these sealing tissue layers vary across seed types. However, it is their chemical composition—particularly the presence and distribution of specific lipids—rather than their physical thickness, that largely determines their functional properties (Borisjuk and Rolletschek, 2009; Munz *et al*., 2017; Langer *et al*., 2023). The formation of these barriers occurs progressively and is tissue specific. MRI can detect not only seed organs, but also characteristic patterns of lipid distribution, offering insights into the spatial organization and functional significance of these internal barriers (Rolletschek *et al*., 2021). In fact, it is capable of non-invasively monitoring the entire development of a plant.

MRI-based analysis of rapeseed development revealed a dynamic pattern of lipid accumulation, initiating in the endosperm and later peaking in the embryo, which becomes the main storage organ (Borisjuk *et al*., 2013b) (Fig. 3). As development proceeds, the outermost endosperm layer (aleurone) synthesizes cuticular compounds and forms a barrier around the embryo (De Giorgi *et al*., 2015). However, lipid deposition is absent in the chalazal region (Fig. 3B), suggesting a site of enhanced solute and gas exchange near vascular tissues (Molina *et al*., 2008), consistent with the phloem unloading zone at the funicular terminus (Pegler *et al*., 2023). MRI further revealed high sugar levels in the peripheral endosperm near the seed coat, and lower levels around the embryo (Rolletschek *et al*., 2021). These 3D imaging insights highlight the endosperm’s role in embryo encasement and assimilate uptake during seed filling.

In the mature seed of rapeseed, the embryo—comprising the radicle, hypocotyl, and two cotyledons—is encased by the testa. The endosperm tightly envelops the embryo, with its lipid-rich aleurone layer situated just beneath the testa. In some regions, the endosperm remains in direct contact with the radicle and the inner surfaces of the cotyledons. This lipid-based envelope acts as a water-isolating barrier that helps regulate the tightly orchestrated process of rehydration and embryonic reactivation during imbibition. The resumption of life—marked by the reinitiation of metabolic activity—begins in the endosperm (Munz *et al*., 2017), in agreement with molecular studies (Bewley *et al*., 2012; Penfield, 2017).

In cereals, seed architecture and function of endosperm are distinct, but the role of the aleurone layer as a barrier appears similar. Lipid accumulation in the aleurone layer is a hallmark of late-stage endosperm differentiation, where the outermost endosperm cells lose their capacity to import nutrients from the apoplast. This developmental transition has significant implications for nutrient allocation, as it restricts the movement of water and assimilates to the inner region of the grain, except the narrow gate in the crease region of the grain (nucellar projection). This pattern is observed in species such as wheat and barley (Radchuk and Borisjuk, 2014). In contrast, members of the *Panicoideae* subfamily, including sorghum (*Sorghum bicolor*) and maize (*Zea mays*), exhibit a different grain architecture. These species form a specialized pedicel region containing a basal endosperm transfer layer, which facilitates the import of assimilates (Wang *et al*., 2012; Sosso *et al*., 2015). Such structural differences between grain types are mirrored by distinct patterns of lipid deposition within the endosperm, as visualized non-invasively using MRI. Our analyses revealed contrasting lipid localizations in the mature grains of representative species from the *Triticeae*, *Bromeae* versus *Brachypodieae*, and *Oryzeae* tribes (Radchuk *et al*., 2019), highlighting the value of lipid imaging for the study of grain filling mechanisms and comparative seed biology in general.

Internal barriers affect gas exchange in living seeds. In maize, the large endosperm is encased by a lipid-rich aleurone layer, which restricts oxygen diffusion. Deep hypoxia becomes the prevailing condition within the endosperm during development (Langer *et al*., 2023). In contrast, the embryo—despite exhibiting a higher respiratory demand—is less affected by such diffusion barriers, owing to its access to oxygen via a basal void space (Fig. 4B). A dedicated study in sunflower (*Helianthus annuus*) seeds further highlighted the relevance of lipid-based barriers in modulating oxygen diffusion. While the thick, multilayered pericarp and attached seed coat contributed little to resistance against gas exchange, a thin, lipid-rich membrane directly covering the embryo was identified as the principal barrier to oxygen diffusion (Borisjuk and Rolletschek, 2009). Comparable scenarios have been reported in other crops, where lipid-containing layers were shown to play a functional role (Rolletschek *et al*., 2025). These findings underscore the importance of internal lipid barriers not only in seed preservation and dormancy, but also in regulating metabolic activity through spatial control of oxygen diffusion.

## Lipid gradients are evident, but what causes them?

Seeds usually consist of genetically distinct organs (embryo, endosperm, testa), each further subdivided into morphological and functional domains and comprising diverse cell types (Radchuk and Borisjuk, 2014; Doll and Ingram, 2022; Pelletier *et al*., 2025). This structural and functional complexity provides a strong basis for spatial heterogeneity in lipid distribution. Such heterogeneity reflects anatomical and physiological features, and it is integral both to the biological functions of individual tissues and to the performance of the seed as a whole.

However, the picture is less clear when looking at lipid gradients within individual otherwise homogeneous tissues. In soybean seeds, for example, dissection of the cellular architecture and genetic circuitry has revealed that most gene co-expression networks are shared across embryonic subregions (Pelletier *et al*., 2025). Identical gene sets, including modules for fatty acid synthesis, are found throughout the soybean embryo. However, steep gradients in local oil storage have been found across cotyledons (Borisjuk *et al*., 2005). Work in rapeseed, for example, has shown that many TAG and phosphatidylcholine species are heterogeneously distributed throughout the embryo (Woodfield *et al*., 2017; Lu *et al*., 2018; Rolletschek *et al*., 2020; Guo *et al*., 2023), and even within each cotyledon significant gradients have been revealed. Similarly high heterogeneity has been described for other oilseeds such as *Glycine max*, *Gossypium hirsutum*, *Camelina sativa*, and *Arabidopsis thaliana* (for review see Borisjuk *et al*., 2023).

This highlights the necessity of addressing numerous open questions. Central issues include how lipid gradients are established during development—whether through genetic regulation, substrate allocation, or biomechanical constraints—and what functional roles they serve at cellular and tissue levels. Do they optimize storage and mobilization, or contribute to traits such as germination, vigour, and longevity? To what extent are lipid gradients stable or temporary, fixed or plastic, and how do they respond to environmental factors or genetic variation? Another key question is how lipid gradients integrate with other spatial patterns, including sugars, proteins, and hormones. Finally, it remains open whether lipid gradients represent conserved evolutionary features or species-specific adaptations, and whether they can be manipulated through breeding or biotechnology to improve oil yield and quality without compromising seed physiology. We now turn to several factors that influence how these gradients are formed.

### Impact of sugar supply and distribution

During the main storage phase, sugars generally reach the embryo via the *trans*-endosperm route (Doll and Ingram, 2022) and can form gradients within the seed (Rolletschek *et al*., 2021; Radchuk *et al*., 2023; Mayer *et al*., 2024). Signalling cascades can translate the local sugar status into metabolic responses, which in turn affect protein stability, enzyme activity, and finally the flux into fatty acids/TAG (Zhai *et al*., 2018, 2021). The molecular mechanisms have only recently been understood: the disaccharide trehalose 6-phosphate (T6P) can signal and regulate sucrose availability (Figueroa and Lunn, 2016). Under high sugar conditions, T6P binds to KIN10, a catalytic subunit of the SUCROSE-NON-FERMENTING1-RELATED PROTEIN KINASE1 (SnRK1). Under these conditions, this central plant metabolic sensor kinase maintains only a basal level of activity, thereby facilitating anabolism (Blanford *et al*., 2024), which includes a high flux into fatty acids. Under low sugar (low T6P) conditions, SnRK1 inactivates several relevant enzyme proteins (such as DGAT1) and transcription factors (such as Wrinkled1), thereby reducing the flux into fatty acids. As SnRK1/KIN10 regulates the phosphorylation status (protein stability) of ∼500 proteins (Artins *et al*., 2024), it is expected that many more regulatory modules will be discovered in the near future. Target of rapamycin (TOR) is another sensor kinase that is activated by both sugars and light and is able to increase the (local) flux into fatty acid synthesis (Liu *et al*., 2025). It is thought that TOR and SnRK1 act antagonistically to maintain the overall balance between anabolism and catabolism. Any shift in the supply of sugars, and thus in local T6P levels, will inevitably affect flux into lipids, and thus may contribute to (transient) lipid gradients. MRI can support integrative approaches by not only monitoring of lipids, but also by visualizing the actual distribution of sugars in tissues using CEST MRI (Mayer *et al*., 2024), detecting the dynamics of ^13^C-labelled compounds (Melkus *et al*., 2011), and revealing structural/functional alterations (Meitzel *et al*., 2021).

### The matter of light supply

Gradients in light availability across tissues lead to gradients in pathway activity. For example, peripheral embryonic tissues of rapeseed can generate most of their ATP/reductant by photosynthesis, whereas inner regions rely entirely on heterotrophic reactions. This locally affects the signalling pathways and contributes to the light-dark cycle, which is known to affect the rate of fatty acid synthesis. Light directly affects TOR kinase as well as acetyl-CoA carboxylase (ACCase) activity by modulating pH (Ye *et al*., 2020a) and other factors. In addition, ‘envelope docking’ of ACCase (Ye *et al*., 2020b) and interaction with the PII protein (sensing cellular carbon and energy availability) may play important roles (Feria Bourrellier *et al*., 2010).

### Effects of allosteric control

Metabolites are often heterogeneously distributed and can therefore locally affect enzyme activities. A variety of local signals/metabolites (e.g. acyl-CoA) allosterically affect ACCase and other fatty acid biosynthetic enzymes, thereby regulating fatty acid synthesis (Andre *et al*., 2012). The same applies to metabolite translocators (Walley *et al*., 2013) and enzyme proteins of the glycolytic pathway upstream of fatty acid synthesis (Schwender *et al*., 2015). The model of bottom-up control of glycolytic flux suggests that increased pyruvate kinase activity lowers the levels of both its substrate phosphoenolpyruvate and its upstream intermediate 3-phosphoglycerate. This in turn deactivates the enzyme ADP-glucose pyrophosphorylase but simultaneously activates phosphofructokinase, thereby increasing glycolytic flux and providing the precursors for fatty acid synthesis (Schwender *et al*., 2015). Allosteric control is generally an important means of flux control in central metabolism.

### Embryo shape and biomechanical aspects

The seed architecture itself shapes embryo metabolism, as the folding of cotyledons during development causes locally different growth conditions for the two cotyledons. This in turn causes metabolic heterogeneity between the cotyledons, as demonstrated and metabolically modelled in Borisjuk *et al*. (2013b). It has also been shown that experimentally induced changes in embryo morphology alter lipid gradients within cotyledons, connected to changes in gene expression (Rolletschek *et al*., 2024).

In summary, the analysis of embryonic cells and seeds based on RNA transcripts, while popular and informative, addresses only one facet of regulation. Post-transcriptional and other mechanisms also play an important role in the regulation of metabolic fluxes in fatty acids/TAGs, whereby lipid gradients can be established. Transcriptionally similar cells can be functionally diverse (and vice versa), as suggested by studies across eukaryotic kingdoms (Özel and Desplan, 2025; Pelletier *et al*., 2025). Genomics can reveal what a seed is capable of, and transcriptomics gives an indication of what the seed intends to do, but the relevant decisions are made *in vivo*. MRI-based analysis of seeds and their lipid gradients can help to reveal this.

## Conclusions and outlook

Seeds and other plant organs are a conglomeration of different tissue and cell types, making spatial heterogeneity an inherent feature. Without recourse to imaging, the task of resolving this heterogeneity is impossible and understanding of its functionality remains limited. MRI is a leading tool for resolving spatial heterogeneity of lipids (Borisjuk *et al*., 2013a, 2023). Although it has lower chemical resolution than mass spectrometry imaging (MSI), it has the unique ability to image tissues in three dimensions and non-invasively *in vivo*. When combined with other multimodal approaches, MRI can address many of the important questions currently at the forefront of plant lipid science.

MRI can be used to identify tissue subregions where oil storage first occurs, as for example in developing embryos, or where oil levels reach a local maximum or remain at a minimum (Borisjuk and Neuberger, 2025). This information can be used to identify and target tissues for dissection-based approaches and comparative omics analyses. Dissection of tissues based solely on visual (anatomical or structural) information can be misleading, as the number of cell identities clearly exceeds the number of anatomically distinguishable cell types (Pelletier *et al*., 2025). In conclusion, defining the gene networks that operate in regions, subregions, tissues, and cell types requires not only comprehensive profiling of all expressed genes, but also prior knowledge of local oil storage activity. Such very specific information can be obtained non-invasively from MRI.

Looking ahead, adapting and applying lipid imaging methods routinely used in clinical MRI may open new avenues for *in vivo* lipid visualization in plants. The most prominent method is the so-called Dixon imaging (Dixon, 1984) and its newer developments (e.g. Melkus *et al*., 2023; Lee *et al*., 2024). So far, it is mainly the heterogeneity of the plant tissue and the additional lipid signals, for example from unsaturated fatty acids, that have prevented researchers from utilizing this method. In the original technique two images with different echo times, one echo time with the water and lipid signal in phase, and one echo time with the water and lipid signal out of phase were acquired. While adding these images results in a water only image, subtracting the two images will lead to a lipid only image. Ultrashort Echo Time (UTE) MRI is not inherently a lipid-imaging technique, but when combined with fat–water separation (e.g. Dixon or spectroscopic approach) could become a powerful approach for imaging short-T_2_ lipid pools (Ma *et al*., 2020; Newbury *et al*., 2025), especially in solid or dry biological systems (like seeds or woody tissues). Conventional spin-echo MRI is often limited in plant research due to rapid T_2_ signal decay, which can make signal acquisition difficult or impossible. UTE MRI, capable of capturing signals in under 1 ms, overcomes this limitation and might significantly broaden MRI’s applicability in plant studies.

Gradients in oil content and composition may be considered a relevant trait for breeding. Separating oil content of different tissues as an independent phenotype for QTL mapping may reveal the genetic mechanism of lipid accumulation (Guo *et al*., 2023). The analysis of MRI-based 3D lipid distribution in seeds using mapping populations could provide new cues (QTLs, genes) to improve oil content. The endogenous heterogeneity of lipid metabolism in oilseeds may be important in designing efficient metabolic engineering strategies to optimise outcomes. Such work may require the use of tissue-specific promoters, which can be obtained from analysis of subregion-specific gene expression (Lu *et al*., 2018; Miray *et al*., 2025; Pelletier *et al*., 2025).

The extent to which apparent lipid heterogeneity is inherited (as opposed to environmental influences) is not fully understood. This is particularly true when considering lipid gradients across a single developing cotyledon or otherwise homogeneous appearing tissues. As outlined above, it is likely that post-transcriptional regulation is relevant to the regulation of lipid metabolism *in vivo*. We need a better understanding of the design principles of lipid metabolism at a spatial and systems level. This in turn requires not only multiple omics-level datasets, but also the determination of metabolic flux as one of the most fundamental metrics of metabolism. To fill this gap, either experimental (Schwender *et al*., 2014; Koley *et al*., 2024; Mukherjee *et al*., 2025) or computational tools (Borisjuk *et al*., 2013; Rolletschek *et al*., 2020) can be applied. The latter also requires experimental constraints derived from ancillary work. MRI could provide relevant information such as tissue-specific growth rates or lipid accumulation rates, that are otherwise difficult to obtain. When further combined with spatial transcriptomics (Yin *et al*., 2023) and MSI-based lipid imaging (Borisjuk *et al*., 2023), metabolic flux analysis could be brought to a spatially resolved scale, opening a new era in lipid science. Determining spatially-resolved metabolic fluxes is the next frontier, and MRI can make an important contribution.

**Fig. 5.**
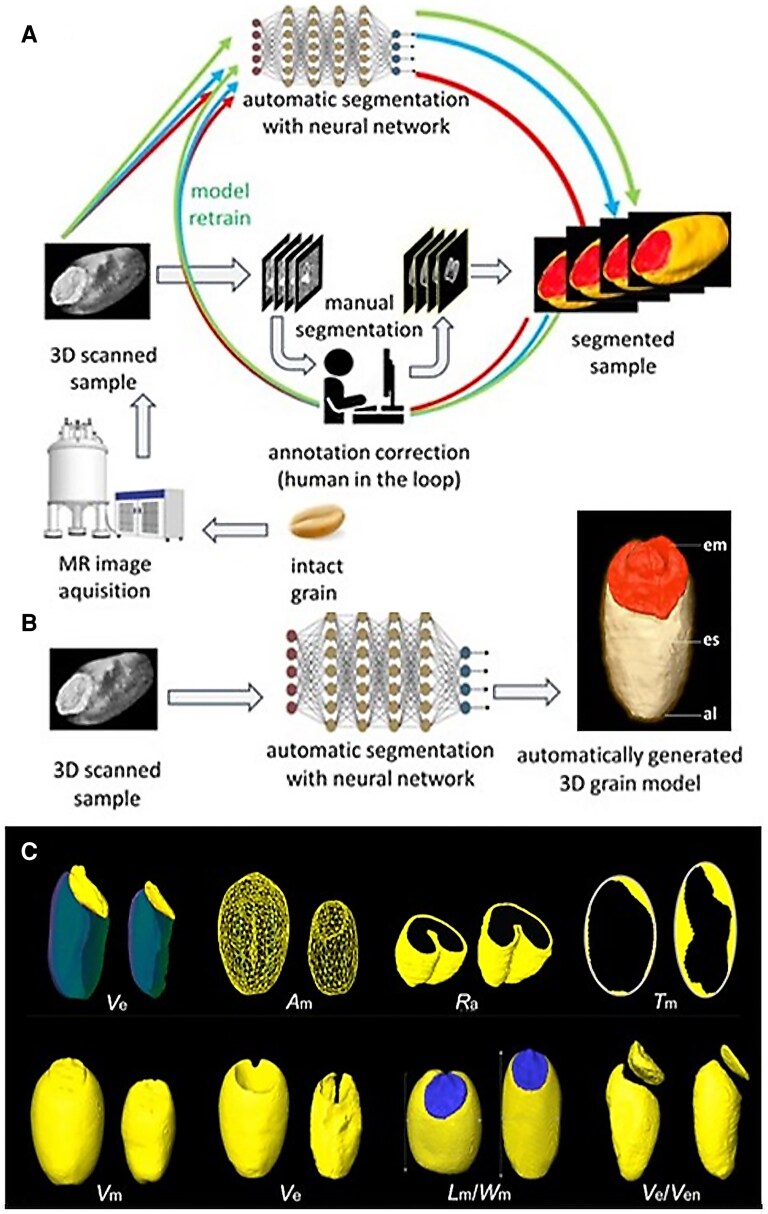
Combining MRI with deep learning algorithms for high throughput seed phenotyping. (A, B) MRI-Seed-Wizard: in the early stages of model development (A), the human expert corrects the predictions made by the pilot model to enrich the training set for the next training iteration—‘human in the loop’. (B) After obtaining a larger dataset, the model is replaced by a 3D variant and the source data are treated as 3D objects producing high-quality segmentation results without human assistance. Abbreviations: al—aleurone; es—endosperm; em—embryo. (C) An example of the MRI-Seed-Wizard application being used on a collection of wheat seeds. Contrasting single-grain morphometrical parameters are shown for two accessions. All images reproduced from Plutenko *et al*. (2025) under a Creative Commons CC-BY license. Abbreviations: Am—surface area of monolith; Lm/Wm—ratio of monolith length to its width; Ra, volume ratio aleurone to monolith; Tm, solidity of monolith; Ve, volume of embryo; Ve/Ven, ratio of embryo volume to endosperm volume; Vm, volume of monolith; Ven, volume of starchy endosperm.

Artificial intelligence (AI) and machine learning are playing an increasingly important role in MRI data analysis, particularly in clinical and plant imaging. To overcome traditional limitations of MRI, such as low throughput and manual data analysis, the integration of deep learning now enables automated, high-resolution phenotyping (Fig. 5). The recently developed MRI-Seed-Wizard tool exemplifies this progress, allowing efficient segmentation and analysis of internal traits in cereal seeds like wheat and barley (Plutenko *et al*., 2025). Lipid-specific signals can be used to improve pattern recognition, tissue segmentation and eventually the number of detectable features. These advances highlight the growing necessity of MRI-based screening for precise, non-destructive seed phenotyping, opening new avenues for crop improvement (Selzner *et al*., 2023; Plutenko *et al*., 2025). Another promising development is Esmraldi, a novel workflow that integrates MRI data with MALDI-Mass Spectrometry Imaging, enabling spatially resolved metabolite mapping in seeds (Grélard *et al*., 2021). Such a multimodal approach can significantly enhance the analytical power of MRI, especially by adding high chemical resolution.

We conclude that MRI of plant lipids represents more than a technical advancement—it is a vital step towards uncovering the metabolic and physiological foundations of plant life, with far-reaching implications for crop improvement. Harnessing MRI for experimental designs in biology often demonstrates that longstanding challenges can be overcome, opening completely new perspectives. For example, the recent discovery that MRI can detect bioluminescence inside living tissues (Ohlendorf *et al*., 2024) enables *in vivo* imaging of bioluminescent molecules in deep tissues which was previously considered nearly impossible.

## Supplementary Material

eraf479_Supplementary_Data

## Supplementary data

The following supplementary data are available at *JXB* online.

Protocol S1. Experimental settings for CSI and CSSI experiments.
