# Supplementary material for: Lipid MRI in plant science: principles and potential areas of application
Source: J Exp Bot. 2025 Oct 28;77(11):3307–22. doi: 10.1093/jxb/eraf479 (PMC13247530; doi:10.1093/jxb/eraf479)
Supplement: eraf479_Supplementary_Data [file eraf479_supplementary_data.pdf]

Supplementary Protocol S1. Experimental settings for CSI and CSSI experiments

The analysis of mixed plant samples with unknown or complex structures—such as fragments of fruits, stems, or roots—as well as highly heterogeneous small organs like seeds, presents significant challenges. In particular, it is often difficult to identify and isolate lipid-storing tissues for detailed lipid fraction analysis using conventional high-resolution techniques. Magnetic Resonance Imaging (MRI) offers a valuable alternative for the initial detection and localization of lipids within intact samples. For optimal results, high static magnetic field strengths and radiofrequency (RF) coils tailored to the sample size are recommended (Webb, 1997).

MRI and MRS experiments using the mixed sample which consisted of relatively large compounds, the freshly hand-cut coconut endosperm and the dried Brazil nut kernel (Fig. 2A-F) were performed using a Bruker Avance Neo 500 MHz Super Wide Bore NMR spectrometer (Bruker BioSpin GmbH, Rheinstetten, Germany) equipped with 1H quadrature birdcage RF resonator with an inner diameter of 66 mm. The proton reference image of the examined 500 µm thick slice was acquired using a standard spin-echo sequence with a repetition time of TR=1000 ms, an echo time of TE= 5.25 ms, eight averages, a field of view FOV of 3.0 x 2.5 cm^2, and a spatial in-plane resolution of 100 × 100 μm^2. The local distribution of water and lipid signals was detected with a slice-selective chemical shift imaging (CSI) sequence applied in spin-echo mode (total measurement time, 3h 57 min). The sequence parameters were as follows: TR=1000 ms; TE=1.05 ms; and slice thickness = 1 mm (same orientation as the reference image). A Hamming-weighted acquisition scheme with 14264 scans was chosen resulting in an effective in plane resolution of 750 × 750 μm^2. The experiment was performed with and without water suppression (VAPOR; suppression pulse bandwidth, 200 Hz). Image reconstruction was performed using MATLAB (MathWorks; https://mathworks.com/). The spectra in this work are shown in absolute mode. The color-coded metabolite images were calculated by integrating the corresponding peak areas of either water or lipids in each voxel.

The analysis of small plant sample organs, such as the oilseed rape (*Brassica napus*) is even more challenging. In order to obtain higher spatial resolution, smaller more sensitive RF resonators and higher static magnetic field strengths are desirable. The following is a brief description of lipid mapping in rapeseed as an example of the processing of intricate samples using CSSI. To shorten the acquisition time a multi-slice multi-echo (MSME) spin echo sequence can substitute the standard 3D spin echo method. In this particular experiment (Fig. 2G-I) small custom-made solenoid coils adjusted to the size of the individual seeds were utilized (Neuberger et al., 2009). The slice thickness was 105 µm and the in plane resolution was 30 × 30 μm^2. A long repetition time (TR = 5 s) was used to avoid the need for T1 correction. Water suppression (to produce a lipid-only image) used a 90° sinc saturation pulse and gradient spoiling before the MSME sequence. The detailed calibration procedure for MRI of *Brassica napus* seed was outlined earlier (Neuberger et al., 2009) and included the tissues segmentation from the images and a reconstruction of the 3D seed structure using commercial software (Amira 4.1), conventional laser microdissection procedure and high-resolution conventional lipid analysis by gas chromatography (Neuberger et al., 2008).

Neuberger T, Rolletschek H, Webb A, Borisjuk L. 2009. Non-invasive mapping of lipids in plant tissue using magnetic resonance imaging. Methods Mol Biol. **579,** 485-96

Neuberger T, Sreenivasulu N, Rokitta M, Rolletschek H, Göbel C, Rutten T, Radchuk V, Feussner I, Wobus U, Jakob P, Webb A, Borisjuk L. 2008. Quantitative imaging of oil storage in developing crop seeds. Plant Biotechnol. J. **6,** 31–45

Webb AG. 1997. Radiofrequency microcoils in magnetic resonance. Prog. NMR Spectrosc. **31,** 1–42.
